# Yeast peptides alleviate diarrhea in neonatal lambs by enhancing the colonic barrier function and modulating colonic microbiota

**DOI:** 10.3389/fvets.2025.1645176

**Published:** 2025-07-28

**Authors:** Dingkun Fan, Rui Zong, Chengrui Zhang, Jixian Zhang, Jianmin Chai, Kai Cui, Naifeng Zhang

**Affiliations:** ^1^Key Laboratory of Feed Biotechnology of the Ministry of Agriculture and Rural Affairs, Institute of Feed Research of Chinese Academy of Agricultural Sciences, Beijing, China; ^2^College of Animal Science, Anhui Science and Technology University, Fengyang, China; ^3^Guangdong Provincial Key Laboratory of Animal Molecular Design and Precise Breeding, School of Animal Science and Technology, Foshan University, Foshan, China

**Keywords:** yeast peptides, lambs, inflammation, barrier function, microbiota

## Abstract

The underdeveloped intestinal tissue and immature microbiota in neonatal lambs predispose to frequent diarrhea or even death, expanding the breeding losses. Yeast peptides are enzymatic products of yeast strains, recognized as antimicrobial peptides due to their demonstrated antimicrobial properties. This study aimed to investigate the impacts of yeast peptides supplementation on the incidence of diarrhea in neonatal lambs, as well as the underlying regulatory mechanisms involved. Thirty-two one-day-old lambs were randomly allocated to four treatments: CON, YP500, YP1000, and YP2000, receiving 0 mg/d, 500 mg/d, 1,000 mg/d, and 2,000 mg/d of yeast peptides, respectively. The dietary supplementation of yeast peptides elicited a significant reduction in fecal scores and the incidence of diarrhea (*p* < 0.05). The administration of yeast peptides to neonatal lambs markedly elevated the levels of anti-inflammatory factors (IL-4, IL-10) while concurrently suppressing the levels of pro-inflammatory factors (IL-1β, IL-6) in the colonic mucosa (*p* < 0.05). Furthermore, yeast peptides enhanced intestinal antioxidant capacity and ultimately strengthened colonic barrier function (*p* < 0.05). Supplementation with yeast peptides altered the colonic microbiota of lambs, characterized by a marked increase in *Roseburia* and a decrease in *Staphylococcus* and *Escherichia_Shigella* abundances. Correlation analysis revealed that the observed attenuation in inflammatory response and enhancement of barrier function were associated with the enrichment of *Roseburia* and the suppression of *Staphylococcus* and *Escherichia_Shigella*. In conclusion, yeast peptides demonstrate potential in ameliorating diarrhea of lambs through the modulation of microbial communities and the enhancement of barrier function in the colon of lambs. The recommended dosage of yeast peptides is 2,000 mg/d.

## 1 Introduction

In recent years, neonatal lamb diarrhea has persisted as a major impediment to the sustainable development of ruminant production systems (1, 2). The immature immune and digestive functions of the intestines create favorable conditions for pathogenic bacterial colonization, which constitutes the primary etiological factor underlying the high incidence of diarrhea in lambs during the initial two-week postnatal period (3, 4). Pathogenic bacteria not only disrupt the establishment of but also produce metabolites capable of translocating across the intestinal epithelial barrier, entering systemic circulation, and directly compromising host health, thereby leading to recurrent diarrheal episodes (2, 5). While antibiotics have demonstrated efficacy in prophylaxis against these conditions, their indiscriminate use has accelerated the emergence of multidrug -resistant bacterial strains, such as enterotoxigenic *Escherichia coli* and *Clostridium perfringens* (6). Addressing this challenge, alternative interventions such as probiotics, organic acids and plant extracts, have been extensively investigated. However, their effectiveness is often constrained by strain specificity and inconsistent performance within the gastrointestinal environment (3, 7, 8). Consequently, the development of new feed additives that possess both broad-spectrum antimicrobial activity and immunomodulatory functions has emerged as a prioritized research direction in contemporary animal nutrition research.

Antimicrobial peptides (AMPs), owing to their broad-spectrum bactericidal activity and efficacy in pathogen eradication, have been developed as dietary additives in farm animal production (9). The multi-target mechanism of action of AMPs imposes evolutionary constraints on the emergence of resistance mutations, positioning them as a promising antibiotic alternative (10, 11). The escalating prevalence of multidrug-resistant bacteria over the past decade has spurred the development and research of AMPs (12). Yeast-derived peptides, generated through enzymatic hydrolysis of yeast cells, have demonstrated beneficial effects on gut health through multiple mechanisms, including direct inhibition of pathogen adhesion (10), modulation of intestinal microbiota (13), and activation of host immune signaling pathways (14). However, the majority of existing studies have primarily concentrated on monogastric animals, leaving critical knowledge gaps regarding their effects and underlying mechanisms in ruminants. Notably, dose-dependence responses may represent a key challenge in optimizing the functionality of yeast peptides (13). Accordingly, we hypothesized that the dietary supplementation with yeast peptides enhances colonic barrier function by promoting a balanced microbiota, which in turn alleviates lamb diarrhea. This research aimed to illustrate how yeast peptides mitigate diarrhea in lambs by assessing their impact on intestinal immunity and microbial barriers, thereby laying the groundwork for enhancing microecological theories in young ruminants and contributing to antibiotic reduction strategies.

## 2 Materials and methods

This trial was carried out on a commercial farm located in FengNing county, Hebei Province, China. The experimental protocol was authorized by the Animal Ethics Committee of the Chinese Academy of Agricultural Sciences (Protocol number: AEC-CAAS-20200515).

### 2.1 Animals, feeding, and trial design

Prior to initiating the formal trial, 41 one-day-old neonatal lambs were subjected to a 14-day fecal scoring assessment to evaluate the prevalence of diarrhea on the experimental farm. Fecal scores were recorded twice daily by two independent observers using a five-point grading system based on criteria previously established (3). The average fecal score was calculated to quantify diarrhea severity. Diarrhea occurrence was calculated daily by identifying lambs with fecal score exceeding 3 (3). The diarrhea occurrence (%) was computed using the following formula: (number of lambs exhibiting diarrhea × number of diarrheic days) / (total number of lambs × total trial days) × 100% (3). The result indicated a diarrhea occurrence of 35.93% and an average fecal score of 2.41, providing essential baseline data for the subsequent experimental phases.

Thirty-two neonatal Hu lambs (4.29 ± 0.22 kg, 1-day-old) of comparable body weight were randomly assigned to four treatments, with eight lambs per treatment. All lambs were suckled by their dams throughout the trial. And the dams in this trial were ensured to be healthy. The treatment groups received yeast peptides supplementation at 0 (CON), 500 (YP500), 1,000 (YP1000), and 2,000 (YP2000) mg/d, respectively. The designated dosage of yeast peptides was uniformly mixed with 10 mL of milk, maintained at 40°C, and administered orally via syringe once daily at 8:00 h. Lambs in the CON group received an equivalent volume of milk without supplementation. The 14-day experimental duration was justified by the brief postpartum period (first two weeks) during which lambs are particularly susceptible to diarrhea and no supplementary feeding is provided, eliminating the need for an adaptation period (3). The pens underwent weekly sanitation to maintain optimal hygienic conditions for both ewes and lambs. Lamb health management and ewe feeding protocols followed standard sheep farm practices. Two lambs from the CON group and one lamb from the YP1000 group were excluded from final analysis due to mortality attributable to insufficient milk intake or diarrhea.

Yeast peptides represent a kind of antibacterial peptide characterized by their unique lasso-like topological structure. This 19-amino-acid-residue peptide, with the specific sequence GGVGKIIEYFIGGGVGRYG, possesses a molecular weight of 1.9 kilodaltons (kD). Notably, yeast peptides maintain a highly stable structural configuration, featuring a distinctive circular form that is intricately folded and contains a lasso running through its core.

### 2.2 Growth performance and diarrhea situation

The trial lambs were weighed on the initial and final days of the trial, after which the average daily gain (ADG) was calculated. Diarrhea incidence was computed as previously described (3).

### 2.3 Serum and colonic mucosa sampling and measurement

On the fifteenth day of the trial, six lambs from each of the four treatments, each selected to approximate the average body weight of its respective group, were designated for slaughter. On the morning of the final day of the trial period, approximately 5 mL of venous blood was collected via jugular venipuncture and subsequently centrifuged at 4°C (3,000 × g for 10 min) to obtain serum for analysis. After slaughter, a 10 cm segment of colonic tissue was excised from the midsection of the colon and cut longitudinally. The colonic luminal contents were rinsed out with phosphate-buffered saline (PBS) and the mucosal layer of the inner colonic wall was scrapped off and transferred into freezing tubes for preservation.

Serum biochemical indicators, including diamine oxidase (DAO) and D-lactase (DLA), as well as biochemical indices of the colonic mucosa, including interleukin 1β (IL-1β), interleukin 4 (IL-4), interleukin 6 (IL-6), interleukin 10 (IL-10), total antioxidant capacity (T-AOC), glutathione peroxidase (GSH-Px), catalase (CAT), superoxide dismutase (SOD) and malondialdehyde (MDA), were assayed using enzyme-linked immunosorbent assay (ELISA) and biochemical kits provided by Nanjing Jiancheng Bioengineering Institute Co., Ltd., Nanjing, China.

After grinding the colon mucosa into powder using a tissue grinder, the tissues were fully lysed by the addition of Trizol. Total RNA from the colon mucosa was then obtained through centrifugation and filtration, and its concentration and purity were then determined with a UV–visible spectrophotometer. After confirming the integrity, complementary DNA (cDNA) was obtained by reverse transcription of the RNA using a cDNA synthesis kit. The resulting cDNA served as the template for quantitative PCR (qPCR) with the primer sequences specified in Table 1. The expression levels of mucoprotein 2 (MUC2), Claudin-1, Claudin-4, Occuludin and Zonula occludens-1 (ZO-1) were quantified using the 2^−ΔΔct^ method, with *β*-actin as the internal reference gene. Each biological replicate was validated in triplicate by qPCR to ensure reliability.

**Table 1:** Target gene, primer sequence and product sizes.

Gene	Primer sequences (5′-3′)	Product size (bp)	Genebank
β-actin	F: CCACAGCCGAGCGGGAAATTG R: AGGAGGACGACGCAGCAGTAG	99 99	XM_004013078.4
MUC2	F: GAGGGCAGAACCCGAAACC R: GGCGAAGTTGTAGTCGCAGAG	131	XM_060404192.1
Claudin-1	F: AACCCGTGCCTTGATGGTGA R: GCCATCCGCATCTTCTGTGC	120 120	NM_001185016.1
Claudin-4	F: TCATCGGCAGCAACATCGTCAC R: CAGCAGCGAGTCGTACACCTTG	110 110	NM_001185017.2
Occludin	F: AGACGCCACGTTGTTGGAGA F: ACAGAGATTTGGCCTCCCGG	107 107	XM_015101255.2
ZO-1	F: ACCATCACGCCAGCATACAATCG R: GCTTTGGAGGACAGGTCAGGTTTG	146 146	XM_015101953.2

### 2.4 Colonic content sampling and 16S rDNA sequencing

After slaughter, the proximal colonic contents were collected and transferred into freezing tubes, which were subsequently stored at −80°C. DNA extraction was performed on the colonic contents using DNA stool kits, resulting in the isolation of genomic DNA. The concentration and purity of the genomic DNA were determined using a Thermo NanoDrop 2000 Ultraviolet Microspectrophotometer (Thermo Fisher Scientific, Waltham, MA), and 1% agarose gel were employed for integrity of quality assessment. Then, targeted amplification of the V3-V4 region of the bacterial 16S rDNA gene was conducted using primers (338F: ACTCCTACGGGAGGCAGCAG; 806 R: GGACTACHVGGGTWTCTAAT). The PCR amplification procedure consisted of an initial denaturation step at 94°C for 5 min, followed by 28 amplification cycles, which included denaturation at 94°C for 45 s, annealing at 55°C for 30 s, and extension at 72°C for 45 s. The process concluded with a final extension step at 72°C for 10 min. The resulting PCR products were purified using AxyPrep DNA gel recovery kits. The constructed library was quantitatively assessed using Qubit and sequencing was performed on the Illumina Miseq PE250 platform, yielding sequenced fragments of 425 bp.

The Fastq sequencing data obtained from Miseq platform were subjected to quality control processing. Subsequently, Qiime2 software was utilized to conduct statistical bioinformatics analysis of Amplicon Sequence Variants (ASVs) at a 100% similarity level. ASV-based analyses determined the diversity and relative abundance of the microflora through summed normalization. The Venn diagram and Mantel test analysis were made with R software (version 4.3.2). The LEfSe analysis was employed to identify the microbial biomarkers across the four treatments. Visualization of co-occurrence network analysis using Cytoscape software (version 3.7.1, Bethesda, MD, USA). Additionally, the relationship between microbial biomarkers and phenotypic parameters was analyzed using a Spearman correlation heatmap in R software (version 4.3.2). PICRUSt2 was utilized for prediction of function compared in comparison with the KEGG database.

### 2.5 Data statistics

Data regarding growth performance, fecal scores, serum indices, and colon biochemical indices were statistically analyzed using the one-way ANOVA procedure by R software (v4.3.2). The incidence of diarrhea was analyzed using the Chi-square test. And the dose-dependent effect of yeast peptides in diet was analyzed by both linear and quadratic regression models. Duncan’s method was employed for multiple comparisons among the four treatments. Microbial abundances were analyzed using the Kruskal-Wallis test to assess differences between the four treatments. Trend plots and column bar charts were visualized by using R software (v4.3.2). A *p*-value of less than 0.05 was considered statistically significant.

## 3 Results

### 3.1 Yeast peptides reduced diarrhea occurrence

As shown in Table 2, there was no significant difference in initial BW among the treatments, satisfying the prerequisites of the trial design. The administration of yeast peptides at varying doses did not significantly affect the final BW or average daily gain (ADG) of the lambs. Nevertheless, the supplementation of yeast peptides to diets significantly reduced (*p* < 0.05) the diarrhea occurrence and the fecal scores of the lambs (Table 2). Specifically, the occurrence of diarrhea was significantly lower (*p* < 0.05) in the YP1000 and YP2000 groups compared to the YP500 group (Table 2).

**Table 2:** Effect of yeast peptides supplementation on growth performance and diarrhea in lambs.

Items	Groups^1^				SEM	*p*-value
	CON	YP500	YP1000	YP2000		
Initial BW, kg	4.29	4.21	4.33	4.26	0.113	0.815
Final BW, kg	7.35	7.28	7.62	7.87	0.406	0.720
ADG, g/d	204.23	212.64	236.79	235.76	6.723	0.476
Fecal score	2.33^a^	1.89^b^	1.91^b^	1.82^b^	0.343	0.005
Diarrhea occurrence, %	32.98^a^	30.36^b^	28.57^c^	28.07^c^	1.044	<0.001

1 CON, control group with 0 g/d yeast peptides; YP500, 500 g/d yeast peptides; YP1000, 1,000 g/d yeast peptides; YP2000, 2,000 g/d yeast peptides; BW, body weight; ADG, average daily gain.

^a,b,c^ Values in the same row with no common letter superscripts mean significant difference (*p* < 0.05).

### 3.2 Yeast peptides decreased colonic inflammatory responses

Supplemental feeding of yeast peptides significantly increased (*p* < 0.05) the levels of IL-1β and IL-6 of the colonic mucosa, while it decreased (*p* < 0.05) the levels of IL-4 and IL-10 (Table 3). No significant differences were observed in the levels of colonic mucosal inflammatory factors between the YP500 and YP1000 lambs. Notably, compared to the YP500 and YP1000 groups, the YP2000 group exhibited higher (*p* < 0.05) levels of anti-inflammatory factors (IL4 and IL10) and lower (*p* < 0.05) levels of pro-inflammatory factors (IL-1β and IL-6).

**Table 3:** Effects of yeast peptides supplementation on colonic inflammatory in lambs.

Items (pg/mg)	Groups^1^				SEM	*p*-value		
	CON	YP500	YP1000	YP2000		Treatment	Linear	Quadratic
IL-1β	14.66^a^	12.82^b^	12.39^b^	11.48^c^	0.269	<0.001	<0.001	0.082
IL-6	39.39^a^	35.21^b^	34.34^b^	32.77^c^	0.610	<0.001	<0.001	0.084
IL-4	9.55^c^	11.40^b^	11.57^b^	11.82^a^	0.206	<0.001	<0.001	<0.001
IL-10	33.92^c^	38.57^b^	39.71^b^	41.01^a^	0.631	<0.001	<0.001	0.016

1 CON, control group with 0 g/d yeast peptides; YP500, 500 g/d yeast peptides; YP1000, 1,000 g/d yeast peptides; YP2000, 2,000 g/d yeast peptides (*n* = 6 per group); IL-1β, interleukin 1β; IL-4, interleukin 4; IL-6, interleukin 6; IL-10, interleukin 10; SEM, standard error of the mean.

^a,b,c^ Values in the same row with no common letter superscripts mean significant difference (*p* < 0.05).

### 3.3 Yeast peptides improved colonic antioxidant capacity

Table 4 presents the levels of antioxidant indices in the colonic mucosa of lambs. The addition of yeast peptides significantly increased (*p* < 0.05) T-AOC, SOD, CAT and GSH-Px levels, while concurrently decreasing (*p* < 0.05) MDA levels in the colonic mucosa. No significant differences were observed in antioxidant indices of the colonic mucosa between the YP500 and YP1000 groups. Among the four treatment groups, the YP2000 group exhibited the highest (*p* < 0.05) levels of T-AOC, SOD, CAT and GSH-Px, while the lowest (*p* < 0.05) level of MDA was also recorded in the YP2000 group.

**Table 4:** Effects of yeast peptides supplementation on colonic antioxidant capacity in lambs.

Items (U/mg)	Groups^1^				SEM	*p*-value		
	CON	YP500	YP1000	YP2000		Treatment	Linear	Quadratic
T-AOC	5.43^c^	7.11^b^	7.26^b^	7.84^a^	0.195	<0.001	<0.001	<0.001
GSH-Px	649.95^c^	727.99^b^	747.02^b^	794.58^a^	12.551	<0.001	<0.001	0.271
CAT	6.63^c^	8.04^b^	8.32^b^	8.89^a^	0.194	<0.001	<0.001	0.037
SOD	74.27^c^	85.71^b^	88.33^b^	92.35^a^	1.482	<0.001	<0.001	0.002
MDA (nmol/mg)	4.20^a^	3.30^b^	3.00^c^	2.71^c^	0.125	<0.001	<0.001	0.006

1 CON, control group with 0 g/d yeast peptides; YP500, 500 g/d yeast peptides; YP1000, 1,000 g/d yeast peptides; YP2000, 2,000 g/d yeast peptides (*n* = 6 per group); T-AOC, total antioxidative capacity; GSH-Px, Glutathione Peroxidase; CAT, catalase; SOD, superoxide dismutase; MDA, malondialdehyde; SEM, standard error of the mean.

^a,b,c^ Values in the same row with no common letter superscripts mean significant difference (*p* < 0.05).

### 3.4 Yeast peptides enhanced intestinal barrier function

Feeding different doses of yeast peptides significantly increased the levels of DAO in serum (*p* < 0.05), and the DAO levels in the serum of YP1000 and YP2000 groups were lower than those in YP500 group (*p* < 0.05). However, yeast peptides did not affect the serum DLA levels of lambs (Table 5). Supplementary yeast peptides significantly upregulated the expressions of MUC2, claudin-4, and Occludin mRNA in colonic mucosa of lambs (*p* < 0.05), whereas the expressions of claudin1 and ZO-1 mRNA were similar across all four groups (Table 5). Moreover, the expression levels of claudin-4 and Occludin genes in YP1000 and YP2000 groups were significantly higher than those in YP500 group (*p* < 0.05), while no differences were observed in the expression levels of MUC2 among the three yeast peptides treatment groups.

**Table 5:** Effects of yeast peptides supplementation on colonic barrier function-related indices in lambs.

Items (ng/mg)	Groups^1^				SEM	*p*-value		
	CON	YP500	YP1000	YP2000		Treatment	Linear	Quadratic
Serum								
DAO	12.15^a^	11.43^b^	10.05^c^	10.43^c^	0.224	<0.001	<0.001	0.004
DLA	8.41	8.57	8.53	8.48	0.075	0.903	0.530	0.737
Colonic mucosa								
MUC2	0.77^b^	1.41^a^	1.49^a^	1.33^a^	0.080	0.001	0.003	0.003
Claudin1	0.81	0.80	0.88	0.95	0.048	0.333	0.272	0.902
Claudin4	1.15^c^	1.87^b^	2.09^a^	2.18^a^	0.131	0.011	0.020	0.159
Occludin	0.59^c^	0.88^b^	1.14^a^	1.41^a^	0.083	<0.001	<0.001	0.897
ZO-1	1.27	1.39	1.48	1.43	0.064	0.326	0.135	0.277

1 CON, control group with 0 g/d yeast peptides; YP500, 500 g/d yeast peptides; YP1000, 1,000 g/d yeast peptides; YP2000, 2,000 g/d yeast peptides (*n* = 6 per group); DAO, diamine oxidase; DLA, Dlactate; MUC2, mucoprotein 2; ZO-1, zonula occludens-1; SEM, standard error of the mean.

^a,b,c^ Values in the same row with no common letter superscripts mean significant difference (*p* < 0.05).

### 3.5 Yeast peptides altered the composition of the colonic microbiota

**Figure 1:**
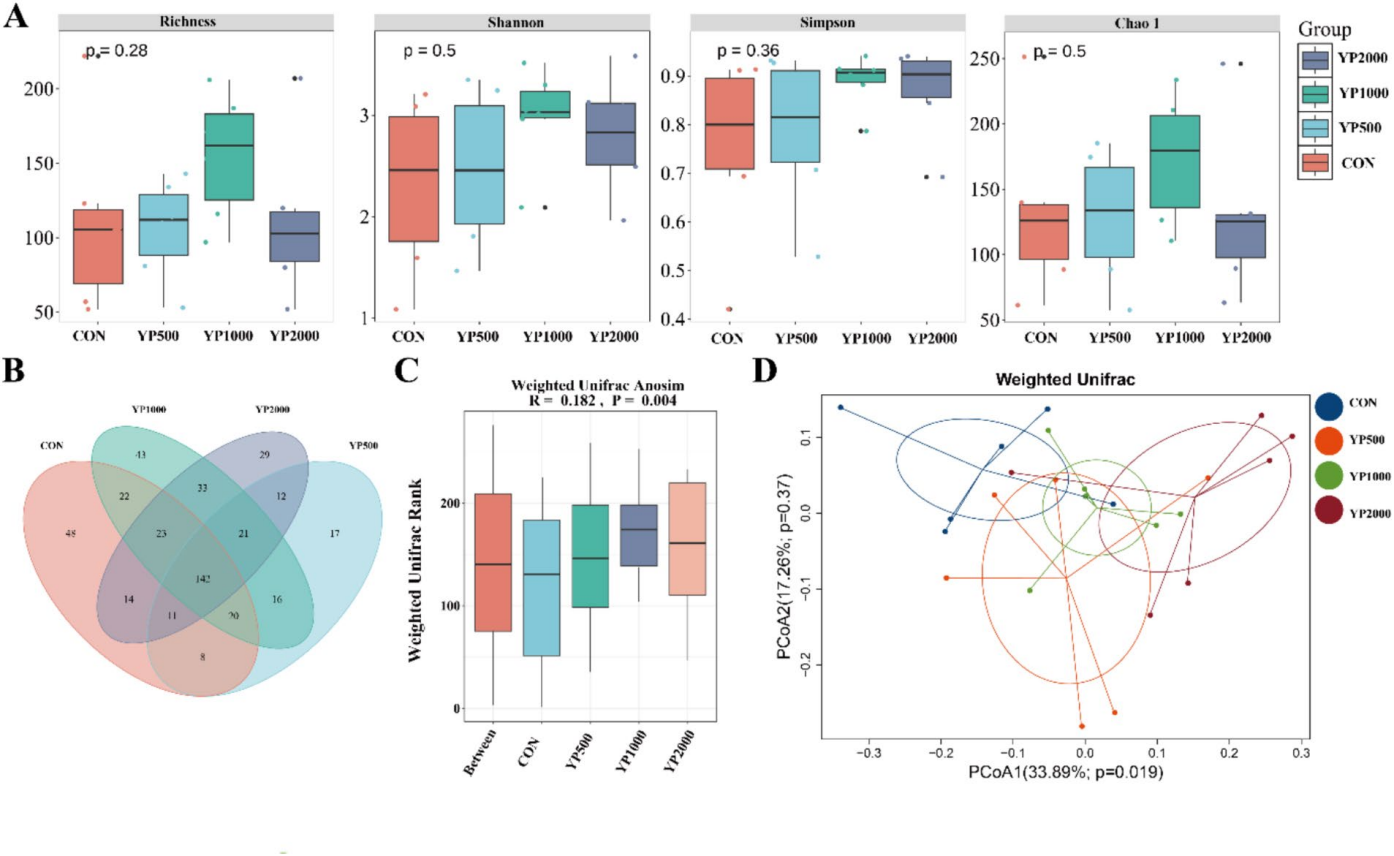
Effects of yeast peptides on microbial diversity. **(A)** Analysis of alpha diversity of colonic microbiota. **(B)** Venn diagrams of the bacterial ASV community. **(C)** Weighted Unifrac Anosim analysis. **(D)** Weighted UniFrac principal coordinate analysis. CON, 0 mg/d yeast peptides; YP500, 500 mg/d yeast peptides; YP1000, 1,000 mg/d yeast peptides; YP2000, 2,000 mg/d yeast peptides, *n* = 6.

**Figure 2:**
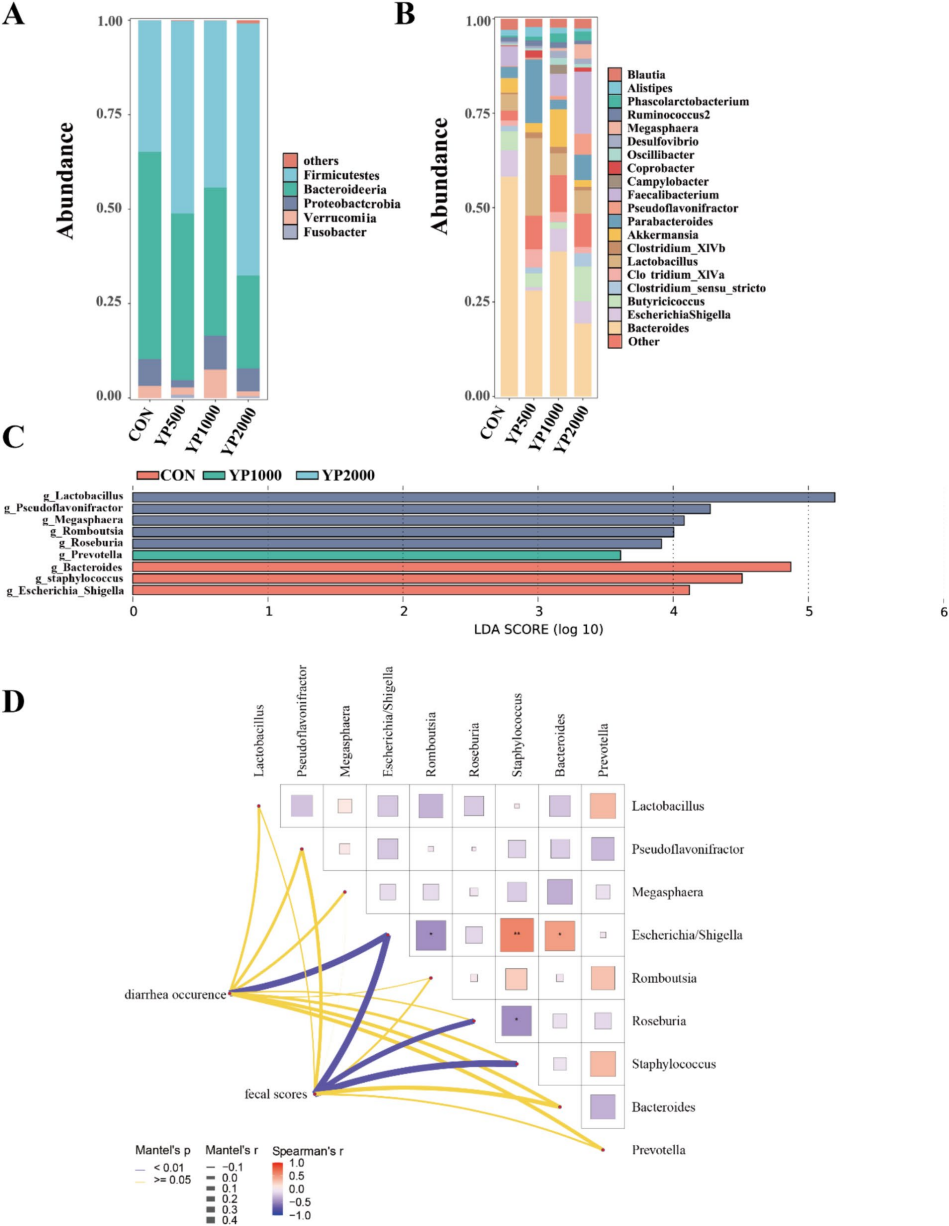
Effects of yeast peptides on gut microbial composition in lambs. **(A)** Relative abundance at the bacterial phylum level in colon. **(B)** Relative abundance at the bacterial genera level in colon. **(C)** Identification of signature bacteria in the colon of four groups of lambs by LEfSe analysis. **(D)** Mantel-test analysis between the signature bacteria and diarrhea situation. CON, 0 mg/d yeast peptides; YP500, 500 mg/d yeast peptides; YP1000, 1,000 mg/d yeast peptides; YP2000, 2,000 mg/d yeast peptides, *n* = 6.

**Figure 3:**
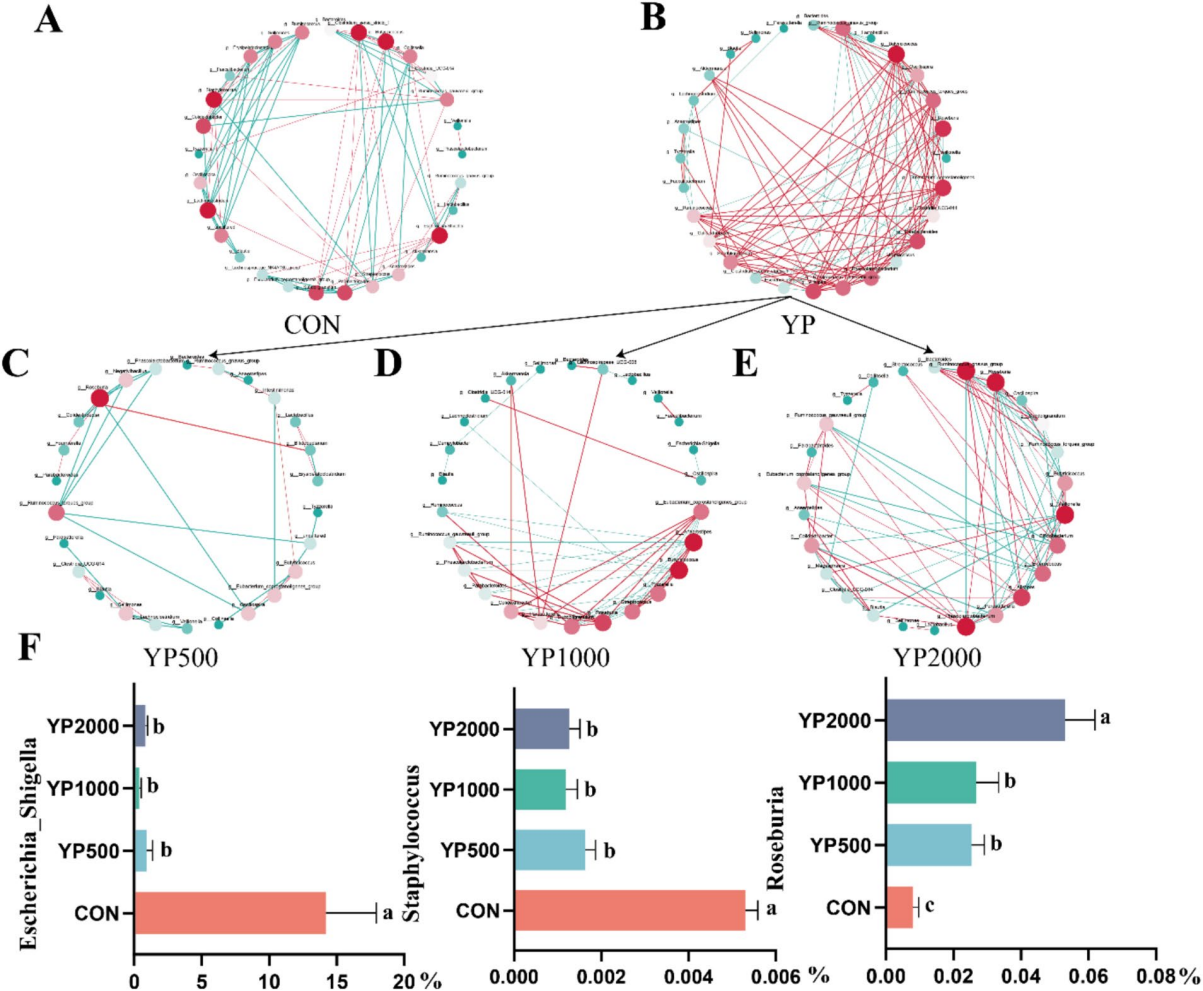
Co-occurrence network analysis identified core microbes among the top 30 genera of colonic microbiota. **(A)** CON group. **(B)** Three yeast peptides supplementation groups. **(C)** YP500 group. **(D)** YP1000 group. **(E)** YP2000 group. **(F)** Relative abundance of core bacterial genera. CON, 0 mg/d yeast peptides; YP500, 500 mg/d yeast peptides; YP1000, 1,000 mg/d yeast peptides; YP2000, 2,000 mg/d yeast peptides, *n* = 6. YP, different doses of yeast peptides supplementation group, *n* = 18.

The sequencing of the 24 samples yielded a total of 358,436,379 clean reads from the colon contents, resulting in the identification of 1,150 ASVs. The four groups exhibited no significant differences in terms of alpha diversity, as measured by Richness, Shannon, Simpson, and Chao1 indices (Figure 1A). Specifically, a total of 288, 247, 320, and 285 ASVs were detected in the CON, YP500, YP1000, and YP2000 groups, respectively (Figure 1B). Furthermore, 142 ASVs were shared among the four groups, while unique ASVs included 48 in CON, 43 in YP500, 29 in YP1000, and 17 in YP2000 (Figure 1B). The Weighted Unifrac Anosim analysis revealed significant differences (*p* < 0.05) between the four groups (Figure 1C). Additionally, the Weighted Unifrac PCoA analysis indicated significant separation between pairs of the four groups (Figure 1D). The colonic microbial compositions among four groups are shown in Figure 2. The ASVs were identified as 8 phyla, and the relative abundance of Firmicutes, Bacteroidetes, Verrucomicrobia, Proteobacteria greater than 1% were considered to be predominant phyla across groups, accounting for 99.32% of the relative abundance (Figure 2A). There were 80 genera detected in all samples. And the predominant genus (relative abundance > 0.5%) included 20 genera across groups, such as *Bacteroides*, *Lactobacillus*, *Parabacteroides*, *Faecalibacterium*, *Escherichia_Shigella*, and *Butyricicoccus*, accounting for 74.36% of the relative abundance (Figure 2B). The LEfSe analysis suggested that the *Escherichia_Shigella*, *Bacteroides* and *Staphylococcus* were significantly enriched in CON group, the *Prevotella* was enriched in YP1000 group, and the *Pseudoflavonifractor*, *Lactobacillus*, *Megasphaera*, *Romboutsia* and *Roseburia* were enriched in YP2000 group (Figure 2C). Based on the mantel test analysis, the abundance of *Roseburia* is negatively correlated with both *Escherichia_Shigella* and *Staphylococcus*, while a positive correlation between the relative abundance of *Escherichia_Shigella* and *Staphylococcus* was observed (Figure 2D). These three genera were significantly associated with diarrhea occurrence and fecal scores in the colon microbiota (Figure 2D). Then, co-occurrence network analysis was conducted to identify the core bacterial genera (R > 0.6; degree cutoff: 1; K-Core: 2) in each treatment group, and the results showed that *Escherichia_Shigella* and *Staphylococcus* were the core bacterial genera in the CON group, while the *Roseburia* was the common core bacterial genera in the YP500, YP1000 and YP2000 groups (Figures 3A–E). Then, the relative abundance of *Staphylococcus* and *Escherichia_Shigella* in YP500, YP1000 and YP2000 groups decreased compared with CON group, while the relative abundance of *Roseburia* increased (Figure 3F).

### 3.6 Yeast peptides changed the colonic microbial function

**Figure 4:**
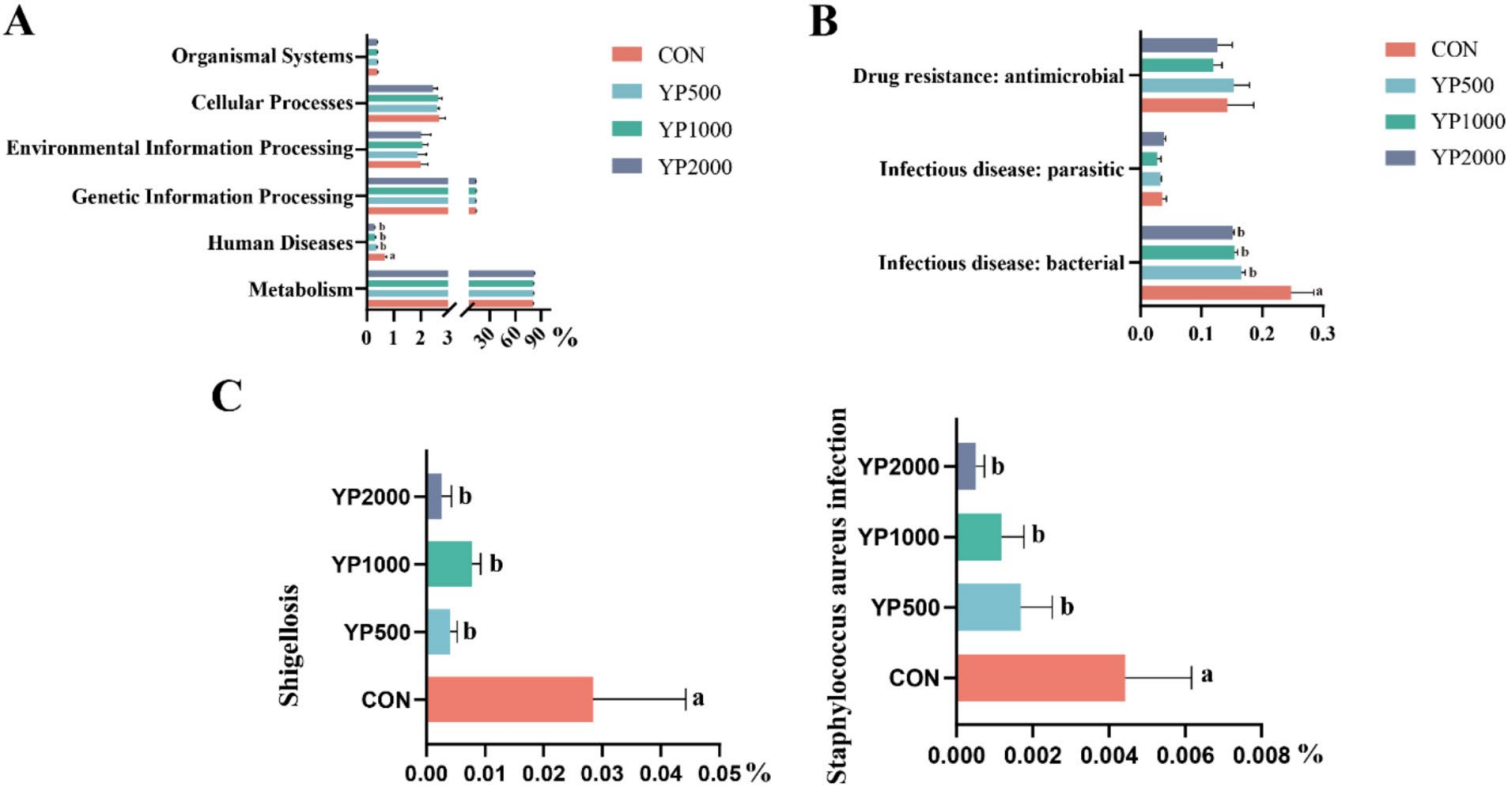
Effects of yeast peptides on comparison of predicted KEGG functions. **(A)** Predicted KEGG differential functions in functional classification level 1. **(B)** Predicted KEGG differential functions in functional classification level 2. **(C)** Predicted KEGG differential functions in functional classification level 3. CON, 0 mg/d yeast peptides; YP500, 500 mg/d yeast peptides; YP1000, 1,000 mg/d yeast peptides; YP2000, 2,000 mg/d yeast peptides, *n* = 6.

**Figure 5:**
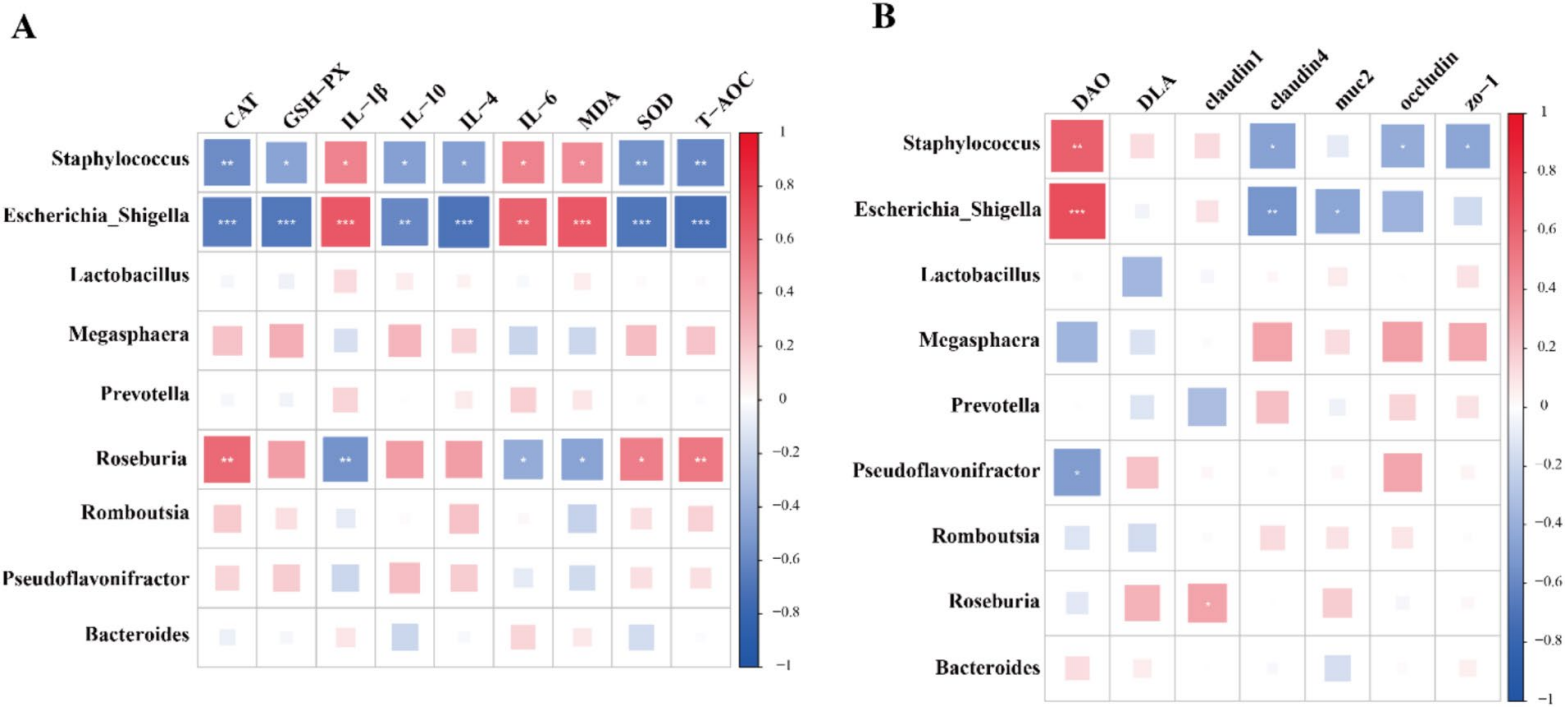
The results of correlation analysis based on spearman correlation coefficient between signature bacteria at the genus level and phenotype parameters. **(A)** Immune and antioxidant functional factors. **(B)** Intestinal barrier functional factors.

Next, the main functional pathways of colonic microbiota were identified based on PICRUSt2 across different treatments (Figure 4). Notably, no significant differences were observed in KEGG level 1 pathways, with the exception of the human diseases pathway. The results indicated that the abundance of human diseases pathway in YP500, YP1000 and YP2000 groups was significantly lower (*p* < 0.05) than that in CON group (Figure 4A). Further analysis of KEGG level 2 pathways of human diseases revealed that only three pathways were detected: drug resistance: antimicrobial, infectious disease: parasitic and infectious disease: bacterial pathways (Figure 4B). Among these, only the infection disease: bacterial pathway in YP500, YP1000 and YP2000 was significantly lower (*p* < 0.05) than the CON group at the second functional level (Figure 4B). The results of the KEGG level 3 pathway analysis indicated that the pathways for Shigellosis and *Staphylococcus aureus* infection were reduced (*p* < 0.05) in YP500, YP1000 and YP2000 groups compared to CON group (Figure 4C). Spearman correlation analysis was conducted for the signature genera and phenotype parameters, which included intestinal immune and antioxidant function (Figure 5). As expected, the microbes *Staphylococcus* and *Escherichia-Shigella* mainly enriched in the CON group exhibited a positive relationship with IL-1β, IL-6, MDA, and DAO. In contrast, these bacteria demonstrated a negative correlation with IL-4, IL-10, CAT, SOD, T-AOC, GSH-Px, and claudin 4 in the colon (Figure 5). Furthermore, *Escherichia_Shigella* displayed a negative correlation with the MUC2, while *Staphylococcus* was negatively correlated with Occludin and ZO-1 (Figure 5). Conversely, *Roseburia* exhibited a positive correlation with CAT, SOD, T-AOC and claudin 1, whereas a negative correlation was observed with MDA, IL-6, and IL-1β (Figure 5).

## 4 Discussion

Diarrhea in neonatal lambs represent a critical health concern that can lead to increased mortality, ultimately adversely impacting both production efficiency and economic profitability (4). Consequently, minimizing the incidence of diarrhea and promoting intestinal health have emerged as paramount objectives in modern sheep production systems. In the present study, although no significant effects on the growth performance of lambs were observed, treatment with different dosages of yeast peptides significantly reduced the occurrence of diarrhea and improved fecal scores.

Gut microbiota serves as a crucial defense mechanism against the invasion of pathogenic bacteria (15). However, neonatal lambs have a rudimentary gut microbiome post-parturition, rendering them vulnerable to harmful bacteria (16, 17). A diverse array of pathogens prevalent in the external environment can colonize the digestive tract of lambs through various routes, potentially leading to diarrhea or even mortality. Previous studies have indicated that AMPs enhance gut health by promoting the proliferation of core bacteria while restricting the growth of pathogens (13, 18). Our findings identified *Roseburia*, *Escherichia-Shigella*, and *Staphylococcus* as key differential genera associated with diarrhea, as determined by Mantel test analysis. *Roseburia* is recognized as a next-generation probiotic due to its capacity to regulate gastrointestinal microbiota, bolster immune responses, and enhance intestinal barrier function (19, 20). Furthermore, the volatile fatty acids produced by *Roseburia* through fermentation of nutrient substrates exhibit a broad range of probiotic effects in animals (19). With increased yeast peptide intake, the abundance of *Roseburia* in lamb colons also increased. When yeast peptides were supplemented in the diet at a level of 2000 mg/d, the abundance of *Roseburia*, *Escherichia-Shigella*, and *Staphylococcus* reached the threshold. Co-occurrence network analysis further confirmed that *Escherichia-Shigella* and *Staphylococcus* were the core genera of the CON group, and *Roseburia* was the core genus of the YP2000 group. However, the co-occurrence relationships in the YP500 and YP1000 groups were not as complex as those in the CON group, while a more complex interaction emerged in the YP2000 group. One possible reason is that medium-dose and low-dose yeast peptides additives inhibited the proliferation of harmful bacteria to a certain extent, and this beneficial effect simplified the co-occurrence network relationship, the benefits of high-dose yeast peptides were optimal, resulting in the formation of a complex co-occurrence network in the colon of lambs in the YP2000 group with probiotics as the core bacteria. Our results demonstrated that the amplification of *Roseburia* elevated immune response to intestinal inflammation and improved the barrier function, which offered additional support to the above study. The adverse effects of excessive colonization of the host intestine by *Escherichia-Shigella* and *Staphylococcus*, including intestinal inflammation and bacterial dysbiosis, are well-documented (21, 22). In this study, the abundance of *Escherichia-Shigella* and *Staphylococcus* were negatively associated with intestinal inflammation factors and barrier function index. Consequently, enhanced gut health appears to be associated with the suppression of *Escherichia-Shigella* and *Staphylococcus* abundance. Furthermore, the downregulation of KEGG functional pathways of Shigellosis and *Staphylococcus aureus* infection further substantiated that yeast peptides modulate the intestinal microflora by inhibiting *Escherichia-Shigella* and *Staphylococcus*. In brief, dietary supplementation with yeast peptides improves intestinal health by regulating the colonic microbiota.

Dysbiosis of intestinal microflora leads to an impaired intestinal barrier. Numerous studies have reported that incorporating AMPs into the daily diet positively impacts gut health in animals, particularly in regulating intestinal barrier function (23). Key components of the physical barrier in the intestinal epithelium include tight junction proteins such as ZOs, claudins, and occludin, which are essential for defending against the invasion of pathogenic bacteria (24). Feng et al. (25) found that administration of cathelicidin-BF resulted in the upregulation of ZO-1, Occludin, and claudin-1 gene expression in the intestine of piglets experiencing diarrhea, thereby strengthening the intestinal barrier function. In another study, it was observed that providing Mastoparan X to mice infected with EHEC O157: H7 significantly increased the levels of ZO-1, Occludin, and MUC2 in both jejunum and colon, consequently enhancing the intestinal barrier function (26). Consistent with these findings, our results demonstrated that the addition of yeast peptides enhanced the colonic barrier function by upregulating expression of genes associated with gut tight junction proteins, such as MUC2, claudin 4 and Occludin, potentially linked to the activation of the mitogen-activated protein kinase (MAPK) signaling pathway (27). Moreover, AMPs regulate the uptake of long-chain fatty acids within intestinal epithelial tissues via the PPAR-*γ* pathway, which is fundamental to the promotion of repair processes in gut epithelial cells (28). Consequently, the enhancement of the barrier function may arise from these regulatory mechanisms. Serum DLA and DAO levels serve as validated biomarkers for assessing intestinal epithelial permeability (29). Notably, our data revealed a significant reduction in serum DAO levels following yeast peptides administration, providing further evidence of their protective effects on intestinal barrier integrity. In summary, dietary yeast peptides exert protective effects against diarrhea by enhancing intestinal barrier function through multiple molecular mechanisms.

Intestinal inflammation induced by dysbiosis of intestinal microbiota is a common complication of diarrhea in lambs (30). The level of inflammatory factors in tissues serve as key indicators of the immune response (31). Yu et al. (32) reported that AMPs Mccj25 upregulated the gene expression of IL-10 in jejunal mucosa of mice, thereby suppressing inflammatory responses. Zong et al. (33) fed LFP-20 to mice with LPS-induced colonic injury and found a significant decrease in the content of IL-6 in colonic tissues. However, several studies have reported opposing results, indicating that certain antimicrobial peptides are able to promote the levels of pro-inflammatory cytokines under specific conditions (25, 34). A possible reason for the discrepancy is that different antimicrobial peptides exert selective modulating effects on the alleviation of inflammation (35). Our results indicated that yeast peptides reduced the levels of colonic pro-inflammatory factors IL-1β and IL-6 while increasing the contents of intestinal anti-inflammatory factors IL-4 and IL-10. These findings suggested that yeast peptides maintain gut health by modulating the gut inflammatory response.

Accumulated studies indicated that alterations in intestinal microbial diversity, particularly the reduction in core bacterial abundance and the proliferation of pathogenic species, are both significant triggers that directly exacerbate intestinal oxidative stress (36, 37). The diminished antioxidant capacity of the intestinal mucosa further compromises the integrity of the intestinal barrier, thereby intensifying the inflammatory cascade, which perpetuates oxidative stress in a self-reinforcing cycle (36). A previous study demonstrated that the administration of yeast peptides to dairy calves enhanced their antioxidant capacity (38). In alignment with these findings, our results suggested that dietary inclusion of yeast peptides contributed to the improvement of intestinal antioxidant capacity in lambs. The appearance of this phenomenon may be associated with modulation of the intestinal microbiota by yeast peptides, which reduced or eliminated LPS and reactive oxygen species (ROS) from intestinal environment (39). Concisely, the dietary supplementation of yeast peptides demonstrates protective effects against diarrhea by enhancing intestinal antioxidant capacity.

## 5 Conclusion

Our findings demonstrated that dietary supplementation with 2,000 mg/d of yeast peptides effectively mitigated diarrhea in neonatal lambs. The yeast peptides modulated the colon microbiota in a prebiotic-like manner by selectively stimulating the expansion of *Roseburia* while restricting the abundance of *Staphylococcus* and *Escherichia_Shigella*. This altered microbiota composition contributed to inhibited colonic inflammation and enhanced colonic barrier integrity, which in turn alleviated the diarrhea symptoms in lambs. These findings elucidated the underlying mechanisms by which yeast peptides enhance intestinal health in lambs and provide a foundation for the application of yeast peptides in young ruminants.

## Data Availability

The original contributions presented in the study are included in the article, further inquiries can be directed to the corresponding author. The datasets used and analyzed during the current study are available from the NCBI Sequence Read Archive (SRA), accession number PRJNA1088740.
